# Colonoscopy Findings: A Single Institution Study from Pakistan

**DOI:** 10.7759/cureus.6167

**Published:** 2019-11-15

**Authors:** Saleh Mohammad, Ghulam Hyder Rind, Iftikhar Ali Shah, Imamuddin Baloch, Azhar Ali Shah, Salma Lakho, Aijaz Ahmed, Aamir Ali Channa, Pinkey Sachdev, Faizan Shaukat

**Affiliations:** 1 Gastroenterology, Ghulam Muhammad Mahar Medical College and Hospital, Sukkur, PAK; 2 Surgery, Ghulam Muhammad Mahar Medical College and Hospital, Sukkur, PAK; 3 Internal Medicine, Ghulam Muhammad Mahar Medical College and Hospital, Sukkur, PAK; 4 Internal Hospital, The Indus Hospital, Rahim Yar Khan, PAK; 5 Internal Medicine, Jinnah Post Graduate Medical Center, Karachi, PAK; 6 Internal Medicine, Jinnah Postgraduate Medical Centre, Karachi, PAK

**Keywords:** colonoscopy, findings, pakistan

## Abstract

Introduction

Colonoscopy is a diagnostic procedure used not only for screening and assessment but also for therapeutic management of various diseases such as removal of polyps, flat lesions, etc. In this study, we determine various outcomes of colonoscopy done in the gastroenterology unit of Ghulam Muhammad Mahar Medical College and Teaching Hospital in Pakistan.

Methods and Materials

This retrospective cross-sectional review was carried out at the colonoscopy unit of Ghulam Muhammad Mahar Medical College and Teaching Hospital in Sukkur, Pakistan. Data was gathered from medical records of patients and by calling their physicians if necessary from July 1 to December 31, 2018.

Results

In our study, the most common site for colonoscopy was a rectosigmoid colon (37.85%, n=134), almost parallel to the anal canal (37.57%, n=133). Normal colonoscopy was reported in 25.42% (n=90). The most common pathology was hemorrhoids (32.48%, n=115), followed by ulcers (17.79%, n=63).

Conclusion

Colonoscopic detection of hemorrhoids was the most common finding in colonoscopy. Normal colonoscopy was less compared to other literature, suggesting physicians are carefully screening patients in advising colonoscopies.

## Introduction

Colonoscopy is a widely established procedure used for screening, assessment, and therapeutic management of colorectal diseases, including polypectomy, removal of flat lesions, dilation, electrocoagulation, and deployment of metal stents for cancerous lesions. It is the gold standard to screen for colorectal cancer and has shown to improve the disease outcome [1].

According to a study published in Iran, the most common outcome of this procedure is normal colonoscopy with a frequency of over 30%, followed by hemorrhoids, polyps, and Infiltrative-exudative wounds [2]. Indications for colonoscopy include average-risk persons over the age of 50 years, family history of colorectal cancer, familial adenomatous polyposis (FAP), hereditary nonpolyposis colon cancer (HNPCC), and blood per rectum or blood in stool [3]. The procedure is associated with various complications. One of the most serious complications associated with colonoscopy is intestinal perforation with leakage of gut contents into the peritoneal space, which has detrimental consequences with a reported mortality rate as high as 5 % [4]. Certain risk factors such as the size of the polyp, recent use of anticoagulants, age, comorbidities, certain drugs including clopidogrel or nonsteroidal anti-inflammatory drugs may influence the outcome of the procedure [5]. 

Previously literature available from Pakistan rarely discusses the findings of colonoscopy in their setting. In this study, we determine the various outcomes of colonoscopy in patients referred to the gastroenterology unit of Ghulam Muhammad Mahar Medical College and Hospital, in the province of Sindh, Pakistan.

## Materials and methods

This retrospective cross-sectional review was carried out at the colonoscopy unit Ghulam Muhammad Mahar Medical College and Teaching Hospital in Sukkur. Data was gathered from medical records of patients and by calling their physicians if necessary from July 1 to December 31, 2018. 

The data retrieved included patient’s age, gender, location and the type of lesion as diagnosed by colonoscopy and pathology. All endoscopic examinations were performed by the gastroenterology faculty of Ghulam Muhammad Mahar Medical College and Teaching Hospital. If indicated, all colonoscopy abnormalities were observed and biopsied. Biopsy and removal were done for all polyps. Bisacodyl 5 mg and sodium docusate 100 mg, six to 10 tablets along with two to three clear-water enemas were given 12 hours before the procedure to prepare the patients. Patients were consciously sedated by giving them 2.5-5 mg of midazolam and 4-6 mg of nalorphine intravenously (IV). After cleaning and washing with standard liquid disinfectant, Olympus® GIF-CF140 (Olympus Corp., Tokyo, Japan) video scopes were used.

Data was analyzed using SPSS version 21.0 (IBM Corp., Armonk, NY). Mean and standard deviation (SD) was calculated for continuous variables such as age. Frequencies and percentages were calculated for categorical variables including gender, location, and outcome.

## Results

During the study period, 369 patients were identified as having a colonoscopy. Out of 369 patients, 15 (4.06%) were excluded due to missing data and 354 were included in further analysis. Among these patients 201 (56.77%) were male and 168 (43.22%) were female. The mean age was 56 ± 17 years (range 13-78 years), while most patients were above the age of 50 years.

The most common site for colonoscopy was a rectosigmoid colon (37.85%), almost parallel to the anal canal (37.57%) (Table 1).

**Table 1:** Sites for colonoscopy

Site	n	%
Recto sigmoid colon	134	37.85%
Anal canal + anus	133	37.57%
Total colon	31	8.76%
Descending colon	30	8.47%
Ascending colon	16	4.52%
Transverse colon	10	2.82%
Total	354	100%

Pathologies were found in nearly 75% of all colonoscopy. The most common pathology was hemorrhoids (32.48%), followed by ulcers (17.79%) (Table 2).

**Table 2:** Findings in colonoscopies

Finding	n	%
Hemorrhoids	115	32.48%
Normal	90	25.42%
Ulcers	63	17.79%
Others	46	12.99%
Polyps	28	7.91%
Fissure	9	2.54%
Diverticulum	3	0.85%
Total	354	100%

## Discussion

All major guidelines advise and recommend colonoscopy for screening in patients older than 50 years. Colonoscopy is also used for colon neoplasm such as familial adenomatous polyposis, hereditary non-polypoid colon cancer, and occult blood in the stool of patients under the age of 50 [6, 7].

In our study, males (56.77%) were the most common patients that underwent colonoscopy. This was consistent with the finding of Betes M et al. and Imperial T et al. who both had more male patients compared to females [8, 9], while Joukar F et al. recorded more female patients compared to males [2].

In our study, 25.42% of participants had no findings in their colonoscopy. This was comparatively lower compared to other studies we found in the literature. Joukar F et al. in his study from Iran reported 35.5% normal colonoscopies [2]. Fernandez E et al. reported normal colonoscopy in normal colonoscopy in 32% [10]. Goners et al. in their study found that only 53% of patients had an appropriate indication for colonoscopy. They found that increasing age and male sex increase the chances of finding significant pathology in colonoscopy [11].

In our study, the most common colonoscopy finding was hemorrhoids (32.48%). This was consistent with the finding of Jaukar F. However, another study conducted in Pakistan, found hemorrhoids in only 10% of patients [12]. The second most common finding was ulcers, followed by polyps. In a study done in Jordan, the most common abnormal findings were colonic cancer in 29%, colonic polyps in 24%, and inflammatory bowel disease in 16% [13]. 

In our study, the most common site for colonoscopy was a rectosigmoid colon (37.85%), followed closely by the anal region (37.57%). This was inconsistent with Joukar F et al. study that reported a higher proportion of colonoscopies performed at anal canal and anus, compared to the rectosigmoid region (43.8% vs 26%).

Our study to the best of our knowledge is the first from the Sindh region, providing useful data regarding pathologies found in colonoscopies. The study has its limitations. Since it was retrospective in nature, how the bowel was prepared was not captured properly. Also, the indications for which colonoscopy was recommended and complications of colonoscopies were not captured.

Pakistan, where a major portion of the population lives in rural areas, there is the evident need for more trained endoscopists. This would help to identify the burden of colonic diseases in our population. Further prospective large scale multi-center trials are needed to consolidate the findings of the limited literature available from Pakistan on colonoscopy findings.

## Conclusions

Hemorrhoids and ulcers were the most common pathological findings in colonoscopies in our study. Almost one-quarter of participants had no pathological findings in their colonoscopies. While normal colonoscopies were less compared to literature from neighboring countries, still efforts should be made to further screen and weigh the risk and benefits before doing the procedure of colonoscopy to avoid unnecessary risk and pain.
